# ASC speck mediated beta‐amyloid seeding in Alzheimer's disease

**DOI:** 10.1002/alz70855_103457

**Published:** 2025-12-23

**Authors:** Michael T. Heneka

**Affiliations:** ^1^ Luxembourg Centre for Systems Biomedicine (LCSB), University of Luxembourg, Luxembourg, Luxembourg

## Abstract

**Background:**

The accumulation of neurotoxic amyloid beta peptides along with neurofibrillary tangle formation are key pathological hallmarks of Alzheimer's disease. The brain has been considered as an immune‐privileged organ, however, increasing evidence from translational, genetic, and pathological studies suggests that activation of distinct innate immune pathways are a third important disease hallmark which actively contributes to disease progression and chronicity.

**Method:**

In vivo 2 photon imaging and in vitro live imaging. Biochemical analysis of protein‐protein interaction.

**Result:**

Microglia play a pivotal role in this immune response and are activated by binding of aggregated proteins or aberrant nucleic acids to pattern recognition receptors. This immune activation leads to the release of inflammatory mediators, but also distracts microglia cells from their physiological functions and tasks. Sustained NLRP3 inflammasome activation in causes a hyperinflammatory microglial cell death called pyroptosis which is the release of ASC specks. The latter contributes to seeding of pathology by enhancing the propensity of beta‐amyloid peptides to aggregate.

**Conclusion:**

This mechanism may account for the spread of pathology within a brain region, but also from one brain area to another. We will provide evidence on ASC speck induced seeding in vivo and in vitro further along with the demonstration of an inhibitory action of biologicals and pharmacological inhibitors.

